# Hydroxychloroquine Potentiates Apoptosis Induced by PPAR*α* Antagonist in 786-O Clear Cell Renal Cell Carcinoma Cells Associated with Inhibiting Autophagy

**DOI:** 10.1155/2021/6631605

**Published:** 2021-04-19

**Authors:** Ruizhe Mao, Jian Shi, Xuyi Ma, Haiyan Xu

**Affiliations:** Laboratory of Urology, The Third Affiliated Hospital of Soochow University, Changzhou 213003, China

## Abstract

Clear cell renal cell carcinoma (ccRCC) is the major pathological pattern of renal cell carcinoma. The ccRCC cells exhibit a certain degree of inherent drug resistance due to some genetic mutations. In recent years, peroxisome proliferator-activated receptor-*α* (PPAR*α*) antagonists have been reported as a targeted therapeutic drug capable of inducing apoptosis and cell cycle arrest in the ccRCC cell line. Autophagy, which can be induced by stress in eukaryotic cells, plays a complex role in the proliferation, survival, and death of tumor cells. In our study, we found that the expression of PPAR*α* was low in highly differentiated ccRCC tissues and 786-O cell line but high in poorly differentiated ccRCC tissues. The level of PPAR*α* expression in ccRCC tissues is correlated to the grade of differentiation, but not to the sex or age of ccRCC patients. The findings also revealed that the PPAR*α* antagonist GW6471 can lower cell viability and induce autophagy in the 786-O ccRCC cell line. This autophagy can be inhibited by hydroxychloroquine. When treated with a combination of hydroxychloroquine and GW6471, the viability of the 786-O cells was decreased further when compared to the treatment with GW6471 or hydroxychloroquine alone, and apoptosis was promoted. Meanwhile, when human kidney 2 cells were cotreated with hydroxychloroquine and GW6471, cell viability was only slightly influenced. Hence, our finding indicates that the combination of GW6471 and hydroxychloroquine may constitute a novel and potentially effective treatment for ccRCC. Furthermore, this approach is likely to be safe owing to its minimal effects on normal renal tissues.

## 1. Introduction

Renal cell carcinoma is one of the malignant tumors derived from normal renal tubular epithelial cells, and clear cell renal cell carcinoma (ccRCC) is its major pathological pattern [1]. According to epidemiological investigations, the morbidity of renal cell carcinoma is ranked second among the various urinary system neoplasms [2]. Metastasis occurs in about 1/3^rd^ of the initially diagnosed renal cell carcinoma patients [3]. Owing to the presence of genetic mutations, renal cell carcinoma can resist traditional chemotherapeutic drugs and radiation [4]. Antiangiogenetic and tyrosine kinase inhibitors, such as sunitinib and sorafenib, have been developed and applied in clinical studies [5, 6]. Unfortunately, the resistance of renal cell carcinoma to these drugs has been reported in recent years [7, 8]. Hence, it is essential to develop novel targeted drugs for renal cell carcinoma.

Peroxisome proliferator-activated receptor (PPAR) is a member of the steroid hormone receptor superfamily that consists of three major subtypes, namely, PPAR*α*, PPAR*β*/*δ*, and PPAR*γ* [9]. PPAR*α* heterodimer with retinoid X receptor (RXR) can bind to peroxisome proliferator responsive element (PPRE) and regulate the expression of several genes associated with lipid, glucose, and amino acid metabolism, as well as inflammatory response [10]. Over the years, researchers have discovered that PPAR*α* may be a potential target in managing leukemia, melanoma, breast cancer, colon cancer, and glioblastomas [11–15]. With regard to ccRCC, Aboud et al. reported that the PPAR*α* antagonist, GW6471, can induce apoptosis and cell cycle arrest [16]. PPAR*α* may be one of the potential targets for treating renal cell carcinoma.

Autophagy is a mechanism in which organelles or macromolecular proteins are “wrapped” into autophagosomes. Small molecules are generated in the lysosomes from the decomposed organelles or macromolecules for recycling by the eukaryotic cells [17]. Autophagy-related genes (ATGs) code most of the autophagy-associated proteins for the initialization of autophagy and the formation of autophagosomes. Autophagic flux is an integral process of autophagy [18]. The basal level of autophagy can regulate proliferation, growth, and differentiation to achieve cellular homeostasis [19, 20]. When cells are in stress, such as during cell cycle arrest, energy deficiency, and growth inhibition, the level of autophagy is enhanced so that they can adapt themselves to the modified conditions and avoid death or other adverse outcomes [21, 22]. The role of autophagy in tumor cell growth and death is unclear, but some researchers believe that it has a protective role by enhancing apoptosis or inducing autophagic cell death, which exerts a “double sword effect” on the fate of the tumor cells [23].

It is unclear whether GW6471 can induce autophagy; besides, its influence on the fate of the ccRCC cells also remains unknown. Therefore, in this study, we attempted to find whether autophagy can be induced in the 786-O ccRCC cell line by GW6471. Furthermore, we tried to ascertain the role of GW6471 in the 786-O cells. We discovered that the expression of PPAR*α* was low in highly differentiated ccRCC tissues and 786-O cells but high in poorly differentiated ccRCC tissues. Subsequently, we came up with evidence that GW6471 can decrease the viability of the 786-O cells and induce integral autophagy. When the 786-O cells were cotreated with GW6471 and hydroxychloroquine, which is an inhibitor of autophagy, the viability was much lower than when treated with GW6471 or hydroxychloroquine alone, and apoptosis was promoted. Moreover, when human kidney 2 (HK-2) cells were cotreated with GW6471 and hydroxychloroquine, there was no significant effect on cell viability. Thus, the combination of hydroxychloroquine and GW6471 may serve as a novel and potentially beneficial strategy for managing ccRCC. Moreover, this approach is likely to be safe as it has only minimal effects on the normal renal tissues.

## 2. Materials and Methods

### 2.1. Cell Lines and Culture

The 786-O ccRCC cell line and the HK-2 human normal kidney tubular epithelial cell line were obtained from the Cell Bank of the Chinese Academy of Science. The two cell lines were cultured in Roswell Park Memorial Institute-1640 medium (Gibco, USA) supplemented with 10% fetal bovine serum (Gibco, USA) and 1% penicillin-streptomycin (Gibco, USA) at 37°C in an incubator (Thermo, USA) with 5% CO_2_ and saturated humidity.

### 2.2. Drugs

The PPAR*α* antagonist GW6471 and the agonist WY14643 were purchased from MCE (USA), and hydroxychloroquine was sourced from Dulai Bio (China).

### 2.3. Immunohistochemical (IHC) Staining

The ccRCC tissues and the adjacent normal tissues of 40 patients were collected from the pathology department of the Third Affiliated Hospital of Soochow University, and all tissues were promptly embedded in paraffin by the staffs. Subsequently, all samples were deparaffinized in xylene and hydrated in a gradient of ethanol (100%, 95%, and 90%). The tissues were then heated in ethylene diamine tetraacetic acid (EDTA) for antigen retrieval and washed thrice with phosphate-buffered saline (PBS), 3 minutes for each wash. Afterwards, Immunohistochemical Staining Kit (Maxim, China) was applied for IHC staining with rabbit anti-human PPAR*α* primary polyclonal antibody (Protech, USA, diluted in 1 : 100) according to the manufacturer's instructions. The tissues were dehydrated in another gradient of ethanol (85%, 95%, and 100%). The nuclei were dyed by hematoxylin. All tissues were covered with microscope slides and observed using a fluorescent optical microscope in the optical mode (Olympus IX77, Japan). The semivalue of the optical density (OD) in IHC was measured using the software ImageJ (USA). The differentiation grade of the ccRCC tissues was analyzed independently by two experienced staffs of the pathology department. The patients' characteristics of age and sex were listed in Table 1. The collection and use of the tissues were approved by the Ethics Committee of The Third Affiliated Hospital of Soochow University.

### 2.4. RNA Extraction and Quantitative Real-Time Polymerase Chain Reaction (qRT-PCR)

Total RNA of the 786-O and HK-2 cells was extracted using the Total RNA-pure Kit (FORE GENE, China) and dissolved in RNase-free ddH_2_O (FORE GENE, China) as per the manufacturer's instructions. Next, the RNA samples were utilized for generating cDNA using the RT Easy™ I Kit (FORE GENE, China). Finally, the cDNA samples were employed for qRT-PCR with the Real-Time PCR Easy™ Kit (FORE GENE, China). The amplification was performed using ABI7500 (Applied Biosystems, USA). The primers of PPAR*α* and GAPDH were synthesized by Takara Biotechnology (Takara, China), and the sequences are listed in Table 2. GAPDH was applied as an internal control, and the relative expression level of PPAR*α* was calculated by the 2^-*ΔΔ*CT^ method [24]. The detailed procedure for qRT-PCR is given in Table 3.

### 2.5. Cell Viability Assay

The 786-O and HK-2 cells were seeded (2∗10^3^) in 96-well plates and incubated for adherence. They were treated on the next day with different groups of drugs for 6, 12, 24, or 48 hours. Then, 10 *μ*L of Cell Counting Kit-8(CCK-8) reagent (Phygene, China) was added to each well. After incubation for 2 hours, the absorbance (*A*) of each well at 450 nm was measured by a microplate reader (Thermo, USA). The percentage of viable cells was calculated using the formula given below:

(1) $$\begin{array}{c} \text{Cell Viability }\left(\%\right)=\frac{\left[A\left(\text{experimental group}\right)-A\left(\text{reagent blank}\right)\right]}{\left[A\left(\text{untreated group}\right)-A\left(\text{reagent blank}\right)\right]}*100\%. \end{array}$$

### 2.6. Acridine Orange Staining

The 786-O cells were seeded (1∗10^4^) in 24-well plates for adherence and treated with different groups of drugs for 24 hours. Before staining, each well was washed with PBS for 15 minutes, three times, and the cells were fixed using 4% poly-formalin for 10 minutes. The dye acridine orange (AO) (Solarbio, China, diluted to 10 *μ*g/mL before staining) was added to each well. After 15 minutes, each well was again washed with PBS for 15 minutes, three times. All the wells were observed using a fluorescent optical microscope in the mode of fluorescence. The nuclei were dyed by AO in green, and the fluorescence of the lysosomes was in orange [25].

#### 2.6.1. Apoptosis Assay by Flow Cytometry

The 786-O cells were collected after treatment and washed twice with PBS. Apoptosis was measured using the Annexin V-FITC/Propidium Iodide (PI) Double Fluorescent Dye Kit (Phygene, China) as per the manufacturer's instructions. The percentage of apoptotic cells was calculated using FACS CantoII Flow Cytometry (BD, USA). Four “gates” were divided by a cross in the images of the flow cytometry: surviving cells were located in the lower left [Annexin V(-) and PI (-)]; early apoptotic cells were located in the lower right [Annexin V (+) and PI (-)]; late apoptotic cells were located in the upper right [Annexin V (+) and PI (+)]; necrotic cells were located in the upper left [Annexin V (-) and PI (+)].

### 2.7. Western Blotting

The collected 786-O cells were treated with drugs for 24 hours and washed twice with PBS. Cell lysis was determined using the Lysis Buffer Kit (KeyGEN, China) on ice according to the manufacturer's instructions. All samples were centrifuged at 4°C and 12000 g/min for 5 minutes. The protein concentration was estimated using the Protein Quantitation Assay Kit (KeyGEN, China) as per the instructions. 5 × loading buffer was added to all the protein samples and heated to 100°C for 5 minutes. The proteins were separated in 8%, 10%, or 15% sodium dodecyl sulfate-polyacrylamide gel electrophoresis. Later, the protein in the gels was transferred to polyvinylidene difluoride membranes (Millipore, USA), and the membranes were blocked with 5% nonfat milk dissolved in Tris-buffered saline (TBST) for 1 hour. When blocking was accomplished, the membranes were incubated with diluted rabbit anti-human primary antibodies by TBST overnight (ABclonal, China, dilution of every antibody: PPAR*α*: 1 : 2000, LC3 : 1: 1000 p62/Sequestosome-1 : 1 : 1000, PARP : 1 : 1000, Caspase-3 : 1 : 1000 GAPDH : 1 : 4000). On the next day, the membranes were washed by TBST for 15 minutes, three times, and incubated with goat anti-rabbit horseradish peroxidase (HRP) conjugated secondary antibody (ABclonal, China, diluted 1 : 8000 using TBST) for 1 hour. After incubating with the secondary antibody, the membranes were washed with TBST for 10 minutes, three times. Finally, the protein signal was detected using the Enhanced Chemiluminescent Plus reagent (Millipore, USA) and visualized by a protein imaging system (Tanon, China). The semivalue of the optical density in Western blotting was measured using the software ImageJ.

### 2.8. Statistical Analysis and Image Capture

Each experiment was performed at least three times independently. All value data were presented as mean ± SEM. The value data were analyzed, and the diagram was drawn using the software Prism 6 (USA). All comparisons of the value data were analyzed using unpaired Student's *t*-test, one-way ANOVA analysis with Fisher Least Significant Difference (LSD) test, and chi-square test in this research of variance. All IHC and fluorescent images were captured by the software Image-Pro-Insight (USA). *P* < 0.05 was considered as a statistically significant difference.

## 3. Results

### 3.1. PPAR*α* Was Poorly Expressed in the Highly Differentiated ccRCC Tissues and 786-O Cell Line but Highly Expressed in the Poorly Differentiated ccRCC Tissues

**Figure 1:**
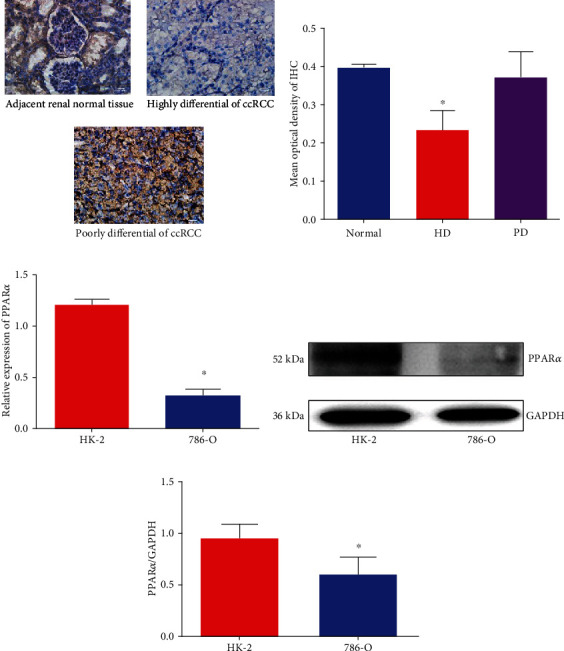
PPAR*α* was poorly expressed in high differentiation of the ccRCC tissues and 786-O cell line, but highly expressed in poorly differentiation of ccRCC tissues. (a) IHC staining for PPAR*α* among different grades of differentiation in ccRCC tissues when compared with the adjacent normal tissues. (b) Mean OD of IHC staining (normal: adjacent normal tissues; HD: highly differentiation; PD: poorly differentiation). (c) The expression of PPAR*α* mRNA in 786-O and HK-2 cells by qRT-PCR normalized by GAPDH. (d) Western blotting for determining the expression of PPAR*α* protein in 786-O and HK-2 cells. (e) Semiquantification of PPAR*α* in 786-O and HK-2 cells normalized by GAPDH (*n* = 3, unpaired Student's *t*-test, ^∗^*P* < 0.05).

In the first part of this research, the expression of PPAR*α* was investigated in the ccRCC tissues and 786-O cell line. As depicted in Figures 1(a) and 1(b), IHC was performed for detecting the expression of PPAR*α* in the ccRCC tissues. The results suggested that the expression of PPAR*α* was lower in the highly differentiated ccRCC tissues than in the adjacent normal tissues. However, PPAR*α* was highly expressed in the poorly differentiated ccRCC tissues, which was similar to that of the adjacent normal tissues. In the 786-O cell line, as illustrated in Figures 1(c)–1(e), we observed that the expressions of the PPAR*α* mRNA and protein were lower when compared with the HK-2 cells, as inferred from qRT-PCR and Western blotting. On the other hand, we investigated the correlation of PPAR*α* expression in ccRCC tissues among some clinical factors, including sex, age, and grade of differentiation. In Table 4, the expression of PPAR*α* in ccRCC tissues was related to the grades of differentiation, but not to sex or age of patients.

### 3.2. GW6471 Decreased the Viability of the 786-O Cells in a Dose-Dependent but Not Time-Dependent Manner, and GW6471 Have No Effect of Cell Viability on HK-2 Cells with Dose-Dependent or Time-Dependent

**Figure 2:**
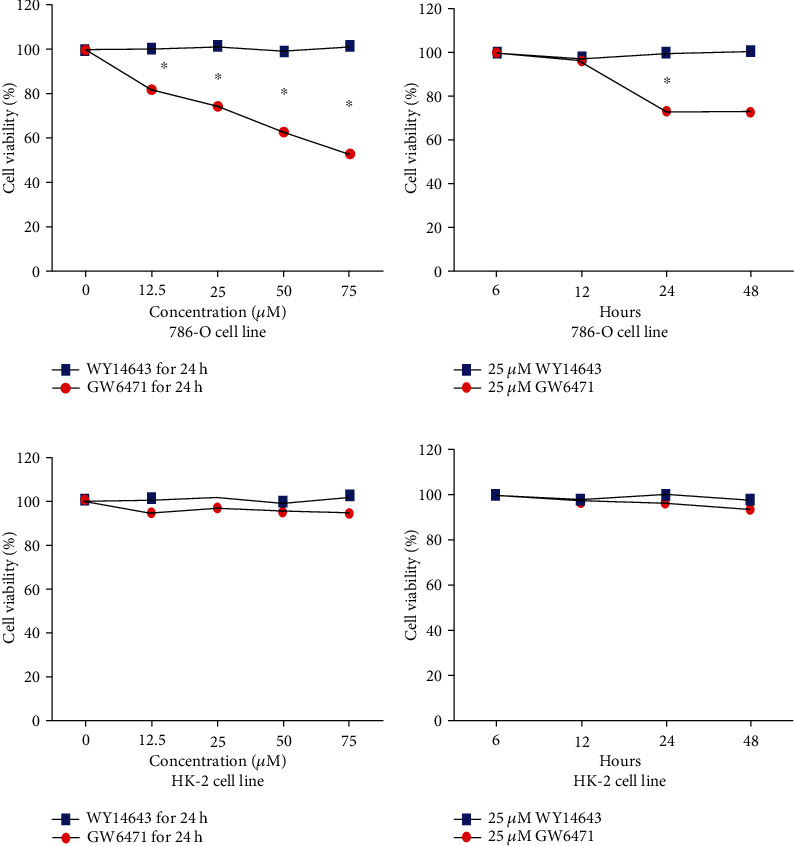
GW6471 decreased the viability of the 786-O cells in a dose-dependent but not time-dependent manner, and GW6471 has no effect of cell viability on HK-2 cells with dose-dependent or time-dependent. (a) Cell viability of 786-O cells treated with different levels of GW6471 and WY14643 (0-75 *μ*M) for 24 h by CCK-8 assay. (b) Cell viability of 786-O cells treated with 25 *μ*M GW6471 or WY14643 for 0, 12, 24, and 48 h by CCK-8 assay. (c) Cell viability of HK-2 cells treated with different levels of GW6471 and WY14643 (0-75 *μ*M) for 24 h by CCK-8 assay. (d) Cell viability of HK-2 cells treated with 25 *μ*M GW6471 and WY14643 for 6, 12, 24, and 48 h by CCK-8 assay. (*n* = 3, unpaired Student's *t*-test ^∗^*P* < 0.05).

Next, we performed a CCK-8 assay for detecting the variations in the viability of the 786-O cells and HK-2 cells treated with GW6471. As shown in Figure 2(a), the viability of the 786-O cells treated with GW6471 decreased in a dose-dependent (0-75 *μ*M) manner. Furthermore, it was noted that WY14643 had no significant effect on the viability of the 786-O cells at any of the tested concentrations (0-75 *μ*M). However, Figure 2(b) demonstrates that when the 786-O cells were treated with 25 *μ*M of GW6471 for 12 hours, there was no significant difference in cell viability when compared with the cells in the 6 h group. On the other hand, when the 786-O cells were treated with 25 *μ*M of GW6471 for 24 hours, the cell viability was lowered when compared with the 6 h and 12 h groups. However, the viability of the 786-O cells treated with GW6471 for 48 hours was not significantly different when compared with those treated for 24 hours. Finally, as shown in Figure 2(c), there was no significant influence on viability in HK-2 cells treated with GW6471 or WY14643 at any of the tested concentrations (0-75 *μ*M); Figure 2(d) demonstrates that when HK-2 cells treated with 25 *μ*M GW6471 or WY14643 for 6, 12, 24, and 48 hours, and there was also no significant effect on cell viability. These results indicated that GW6471 can decrease the viability of the 786-O cells in a dose-dependent but not time-dependent manner, and GW6471 has no effect on cell viability in HK-2 cells with dose-dependent or time-dependent.

### 3.3. GW6471 Induced Integral Autophagy in 786-O Cells

**Figure 3:**
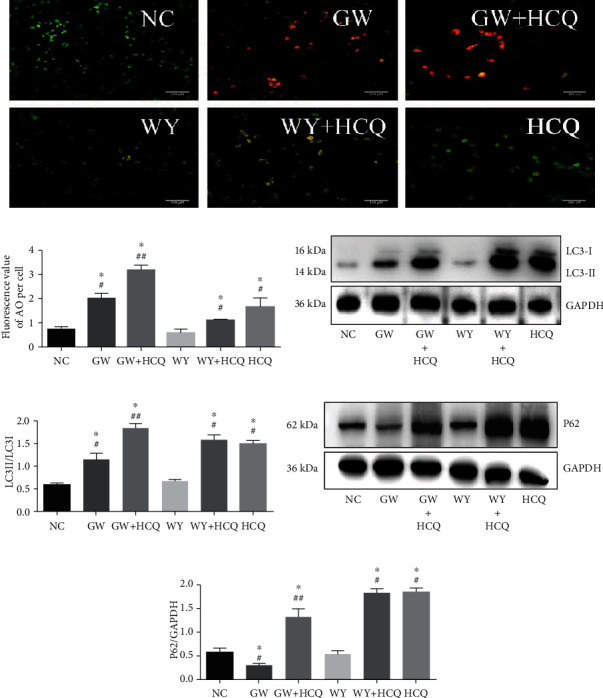
GW6471-induced integral autophagy in 786-O cells. (a) Images of AO dyed in 786-O cells treated with different groups of drugs. (b) Semivalue of AO's fluorescent signal in 786-O cells treated with different groups of drugs normalized to per cell. (c) The expression of LC3-I/II was analyzed by western blotting. (d) Ratio of LC3-II/LC3-I. (e) Expression of p62 was analyzed by western blotting. (f) Semiquantification of p62 normalized by GAPDH. (25 *μ*M GW6471, WY14643, and 50 *μ*M hydroxychloroquine was applied in validating autophagic flux and the remaining experiment. (*n* = 3, one-way ANOVA with LSD ^∗^*P* < 0.05, ^#^compared with untreated group (NC), ^##^compared with the group of GW6471).

GW6471 can decrease cell viability and induce cell cycle arrest and apoptosis in the 786-O cells; hence, we investigated whether GW6471 can induce autophagy in the 786-O cells [16]. Based on existing references about other human tumors treated with GW6471 and our own preliminary experiments, we chose 25 *μ*M of GW6471 and WY14643 and 50 *μ*M of hydroxychloroquine to treat the cells for a duration of 24 hours for the rest of the experiments in this research [26]. Hydroxychloroquine (or chloroquine, which was applied in some studies on autophagy) is an autophagy blocker that can inhibit the fusing of autophagosomes with lysosomes [27]. We first utilized AO for dyeing the lysosomes, as shown in Figures 3(a) and 3(b), and discovered that the fluorescent signal of the lysosomes normalized to per cell was brighter in the group treated with GW6471 than in the untreated and WY14643 treated groups. In the group treated with GW6471 and hydroxychloroquine, the fluorescent signal was much brighter than that in the group treated with GW6471 or hydroxychloroquine alone. As shown in Figures 3(c) and 3(d), the ratio of LC3-II/LC3-I, which is considered as a marker of autophagy induction and analyzed by Western blot, was increased in the GW6471 group when compared with the untreated and WY14643 groups. In the GW6471 with hydroxychloroquine group, the ratio of LC3-II/LC3-I was the highest among all the experimental groups. p62 (sequestosome-1, SQSTM-1), a protein which can be used to monitor autophagy flux, was found to decrease in the GW6471 alone group but increase in the GW6471 with hydroxychloroquine group, as shown in Figures 3(e) and 3(f) [27]. The results of AO dyeing and Western blotting indicated that GW6471 induced integral autophagy in the 786-O cells.

### 3.4. On Cotreatment of GW6471 and Hydroxychloroquine, the Cell Viability of 786-O Cells Was Further Decreased

**Figure 4:**
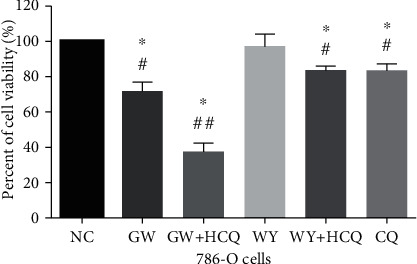
On cotreatment of hydroxychloroquine and GW6471, the cell viability of 786-O cells was decreased further (CCK-8 assay). (*n* = 3, one-way ANOVA with LSD ^∗^*P* < 0.05, ^#^compared with untreated group (NC), ^##^compared with the group of GW6471).

Autophagy can affect the fate of the tumor cells; hence, we explored whether inhibiting autophagy can alter the viability of the 786-O cells treated with GW6471 subsequently. As presented in Figure 4, cotreatment with GW6471 and hydroxychloroquine for 24 hours decreased the viability of the 786-O cells more profoundly than the treatment with GW6471, hydroxychloroquine alone, and WY14643 with hydroxychloroquine.

### 3.5. On Cotreatment of Hydroxychloroquine and GW6471, the Ratio of Apoptotic Cells Was Promoted in 786-O Cells

**Figure 5:**
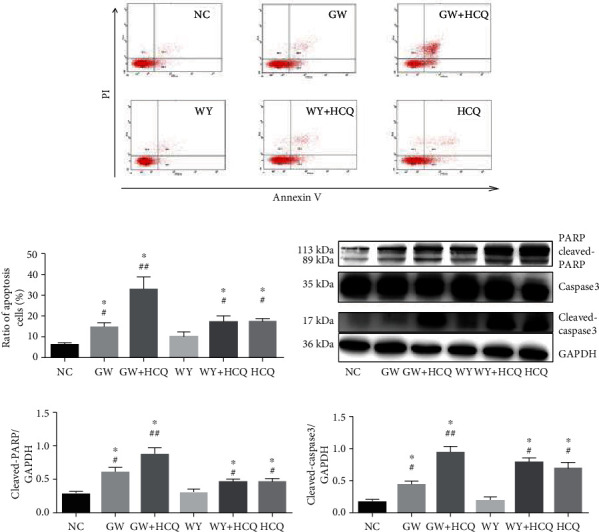
On cotreatment of hydroxychloroquine and GW6471, the ratio of apoptotic cells was promoted in 786-O cells. (a) Flow cytometry was performed for detecting apoptotic cells. (b) Ratio of apoptotic cells was calculated by flow cytometry. (c) The expression of cleaved-PARP and cleaved caspase-3 was analyzed by western blotting. (d) Semiquantification of cleaved-PARP normalized by GAPDH. (e) Semiquantification of cleaved caspase-3 normalized by GAPDH. (*n* = 3, one-way ANOVA with LSD ^∗^*P* < 0.05, ^#^compared with untreated group, ^##^compared with group of GW6471).

The viability of the 786-O cells treated with a combination of GW6471 and hydroxychloroquine was lower than that of the cells treated with GW6471 or hydroxychloroquine alone. We investigated the mechanism of death in the 786-O cells treated with the combination of GW6471 and hydroxychloroquine. Flow cytometry was performed, and as shown in Figures 5(a) and 5(b), the ratio of apoptotic cells in the GW6471 group was higher than that in the untreated and WY14643 with or without hydroxychloroquine groups. When the 786-O cells were cotreated with GW6471 and hydroxychloroquine, the ratio of the apoptotic cells was higher than that in the cells treated with GW6471 alone. Subsequently, we performed western blotting for detecting cleaved-PARP and cleaved caspase-3, which are the two markers of cellular apoptosis. As inferred from Figures 5(c)–5(e), the expressions of cleaved PARP and cleaved caspase-3 in the GW6471, hydroxychloroquine alone, and WY14643 with hydroxychloroquine groups were higher than those in the untreated and WY14643 alone groups. When the 786-O cells were co-treated with GW6471 and hydroxychloroquine, the expressions of cleaved PARP and cleaved caspase-3 were the highest among all the experimental groups. The results displayed in Figure 5 indicate that hydroxychloroquine potentiated apoptosis in the 786-O cells treated with GW6471.

### 3.6. On Cotreatment of GW6471 and Hydroxychloroquine, HK-2 Cells Showed No Significant Effect on Cell Viability

**Figure 6:**
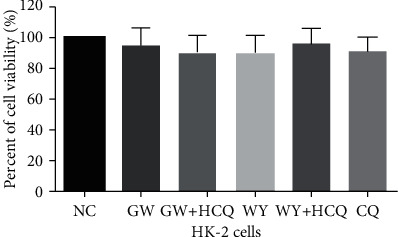
On cotreatment of GW6471 and hydroxychloroquine, the HK-2 cells showed no significant effect on cell viability. (*n* = 3, CCK-8 assay).

We finally explored whether GW6471 combined with hydroxychloroquine can influence the viability of the HK-2 cells. As indicated in Figure 6, when the HK-2 cells were treated with the drugs for 24 hours, there was no significant effect on cell viability in any of the experimental groups, including the group of GW6471 with hydroxychloroquine. This result illustrates that cotreatment with GW6471 and hydroxychloroquine might not have a significant cytotoxic effect on the HK-2 cells.

## 4. Discussion

PPAR*α* can regulate nutrient metabolism as a transcription factor. In hepatocytes, PPAR*α* is highly expressed and can regulate carnitine palmitoyltransferase 1A and medium chain acyl-coenzyme A dehydrogenase, thereby enhancing fatty acid *β*-oxidation [28]. In the metabolism of glucose, PPAR*α* heterodimer with RXR can bind to the genes encoding phosphofructokinase-1 (PFK-1) and pyruvate kinase (PK) with the sequence promoter, thus, regulating the expression of these two enzymes [29]. Additionally, in PPAR*α*^−/−^ mice, arginine is accumulated in the plasma and the level of nitric oxide (NO) is lowered [30], which implies that PPAR*α* may influence amino acid metabolism. The expression of PPAR*α* in the ccRCC cells has been researched in the past. In the present investigation, our results demonstrated that the expression of PPAR*α* was low in the highly differentiated ccRCC tissues and the 786-O cell line but high in the poorly differentiated ccRCC tissues. The predominant morphological feature of the ccRCC cells is the presence of abundant lipid droplets in the cytoplasm [31]. Accumulation of lipid droplets in the ccRCC cells is associated with several genes, including the underexpression of carnitine palmitoyltransferase 1A (CPT1A) and the overexpression of the fatty acid synthase (FASN), perilipin-3 [32–34]. Moreover, Du et al. reported that the accumulation of lipid droplets in the 786-O cells is associated with the low expression of CPT1A, and glucose is the major ingredient for the formation of the lipid droplets in the 786-O cells [35]. However, Wettersten et al. reported that CPT1A may catalyze the synthesis of acylcarnitines from free fatty acids for anti-inflammatory action in high-grade ccRCC [36]. The CPT1A gene can be regulated by PPAR*α* [37]. Based on our research of PPAR*α* expression in different grades of ccRCC tissues and previous studies by other investigators, it could be stated that PPAR*α* might exert complex and variable effects on proliferation and growth in different grades of ccRCC. Finally, as the results of Table 4, the expression of PPAR*α* in ccRCC tissues was correlated to the grade of differentiation, but not to the sex or age of patients. PPAR*α* may be a potential marker for differentiating grades of ccRCC tissues. In the future, more samples of ccRCC tissue will be collected for investigating the correlation between the expression of PPAR*α* in ccRCC and clinically relevant profiles.

The role of PPAR*α* in the treatment of different tumors is also complex and variable. PPAR*α* is poorly expressed in melanoma, and fenofibrate (an agonist of PPAR*α*) can inhibit the metastasis of melanoma via the downregulation of AKT and ERK1/2 phosphorylation [38]. PPAR*α* is highly expressed in paraganglioma. When the paraganglioma cell lines were treated with GW6471, cellular apoptosis was promoted and migration was inhibited via the repression of the PI3K/GSK3/*β*-catenin pathway [26]. On the other hand, PPAR*α* is overexpressed in pancreatic cancer tissues when compared with their adjacent normal tissues. However, clofibrate (another PPAR*α* agonist) can induce apoptosis and sensitize the cells to radiation through the downregulation of the Wnt/*β*-catenin pathway. In the ccRCC cells, glycolysis is the major pathway for energy generation. GW6471 can block glycolysis and induce cell cycle arrest via the downregulation of the oncogene c-Myc [39]. In this research, we discovered that although PPAR*α* was poorly expressed in the 786-O cells when compared with the HK-2 cells, the viability of the former cells can be decreased by GW6471 in a dose-dependent but not time-dependent manner. Our results are similar to those obtained by Aboud et al. [16]. However, we discovered that WY14643 has no significant effect on the viability of the 786-O cells. Based on the metabolism of ccRCC, we speculated that the low expression of PPAR*α* may be essential for maintaining the basal level of glycolysis in the 786-O cells. The reason for WY14643 not having a significant effect on the viability of the 786-O cells remains to be explored in future studies.

In renal cell carcinoma, the basal level of autophagy plays an important role in cellular growth and proliferation. Ma et al. applied IHC and discovered that the expression of ATG9 is higher in the ccRCC tissues than in the adjacent normal tissues and that this elevated expression is an independent risk factor of prognosis in ccRCC [40]. Lu et al. reported that UNC-51-like kinase 1 (ULK1, ATG1) is another autophagy-associated protein and that the high expression of ULK1 is an independent risk factor of prognosis in ccRCC [41]. When cells are under stress, autophagy can be induced via the suppression of thePI3K/AKT/mTOR and MAPK/ERK/mTOR pathways or the activation of the AMPK/mTOR pathway [42–44]. Based on the findings of Aboud et al. and ours, we further explored whether autophagy can be induced by GW6471 in the 786-O cells. AO is a fluorescent dye that can stain the acidic vesicular organelles in red and the nuclei in green [25]. Hydroxychloroquine is a weakly alkaline drug that can slightly increase the pH of the lysosomes, inhibit autophagosome-lysosome fusion, and induce an autophagy-independent severe disorganization of the Golgi and endo-lysosomal systems, which may cause fusion impairment [45]. When autophagy is induced in the cells and the autophagic flux is fluent, the AO signal can be detected significantly in the cells. Since the autophagic flux is blocked by hydroxychloroquine, lysosomes and autophagosomes can be accumulated and observed in the cells [46]. We discovered that when the 786-O cells were treated with GW6471 alone, the fluorescent signal of the lysosomes dyed with AO was brighter than that of the untreated and WY14643 groups. When the cells were treated with a combination of GW6471 and hydroxychloroquine, the fluorescent signal was significantly brighter and more abundant than that of the GW6471, hydroxychloroquine alone, and WY14643 with hydroxychloroquine groups. This observation proves that lysosomes are more in the 786-O cells treated with GW6471 than in the untreated and WY14643 groups and that they are accumulated in the GW6471 with hydroxychloroquine group. LC3 (ATG8) is a marker of autophagosomes, and it includes two types, namely, LC3-I and LC3-II. LC3-II is the lipidation type of LC3-I, which is catalyzed by ATG4 and is considered as a marker of mature autophagosomes [47]. p62 (Sequestosome 1/SQSTM1) is a 62 kDa protein that is involved in cell survival, apoptosis, inflammation, and autophagy [48]. Two domains of p62 are associated with autophagy. The UBA domain can bind to the ubiquitinated protein, and the LIR domain can interact with LC3 on the surface of the autophagosomes. Finally, p62 is degraded together with its cargo in the autophagosomes fused with the lysosomes when the autophagic flux is fluent [49, 50]. However, when the autophagic flux is blocked, p62 will be accumulated in cells [51]. By western blotting, our research demonstrated that the ratio of LC3-II/LC3-I in the GW6471 group was higher than that in the untreated and WY14643 groups. In the GW6471 with hydroxychloroquine group, the ratio of LC3-II/LC3-I was higher than that in the GW6471, hydroxychloroquine alone, and WY14643 with hydroxychloroquine groups. Meanwhile, the expression of p62 in the GW6471 group was lower than that in any other groups, and p62 was accumulated in the GW6471 with the hydroxychloroquine group. These results indicate that GW6471 can induce integral autophagy in the 786-O cells and that the autophagy can be blocked by hydroxychloroquine.

**Figure 7:**
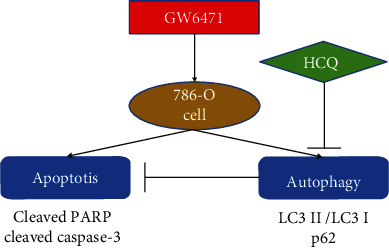
Model of synergistic antitumor effects of GW6471 and hydroxychloroquine in 786-O cells. GW6471 can induce apoptosis and autophagy in 786-O cells. Hydroxychloroquine can inhibit autophagy induced by GW6471 in 786-O cells. Apoptosis can be potentiated in 786-O cells cotreated with GW6471 and hydroxychloroquine compared with being treated GW6471 only.

The role of autophagy in the tumor cells is still unclear. In renal cell carcinoma, autophagy exerts a dual effect on the fate of the cells [52]. Sunitinib, a first-line drug which is an inhibitor of tyrosine kinase, can block angiogenesis and inhibit cellular proliferation in the treatment of renal cell carcinoma [53]. Li et al. uncovered that this drug can decrease cell viability in the OS-RC-2 renal cell carcinoma cell line and induce autophagy via the downregulation of the PI3K/AKT/mTOR pathway. When the OS-RC-2 renal cell carcinoma cells were cotreated with sunitinib and chloroquine, the decrease in cell viability was more than that of the cells treated with sunitinib alone, which could be attributed to the promotion of apoptosis [54]. Mammalian target of rapamycin a (mTOR) is a sensor of nutrition and cellular growth, and it consists of two subtypes, namely, mTOR1 and mTOR2. mTOR can regulate autophagy as a key protein by “perceiving” the variations in the upstream signals of PI3K/AKT, MAPK/ERK, and AMPK [55]. In renal cell carcinoma, the expression of mTOR is higher than that in the normal renal tissues and is therefore acclaimed as a target in the treatment of RCC [56]. Zheng et al. reported that the mTOR dual inhibitor AZD-2014 can inhibit cell growth and induce autophagy in the 786-O and OS-RC-2 cells. In the two cell lines treated with a combination of AZD-2014 and chloroquine, cell viability was decreased further by the promotion of apoptosis [57]. This finding implies that AZD-2014 and chloroquine may have a synergistic effect in killing renal cell carcinoma cells. Autophagy induced by drugs in renal cell carcinoma may enhance cellular death. Ubenimex, an inhibitor of aminopeptidase, can enhance immunity and is considered as a potential drug for treating renal cell carcinoma [58]. Ubenimex can induce autophagy in the 786-O and OS-RC-2 cells, and when the two cell lines were cotreated with ubenimex and rapamycin (an inhibitor of mTOR), cell growth was inhibited. However, when 3-methyladenine (3-MA), another autophagy blocker, was combined with ubenimex, there was no significant difference in cell viability when compared with ubenimex alone [59]. This result alludes that the autophagic flux enhanced by ubenimex is correlated with cell death in renal cell carcinoma. Sorafenib is another inhibitor of angiogenesis and tyrosine kinase and is also applied for treating renal cell carcinoma [60]. The drug can inhibit cellular growth and induce autophagy via the downregulation of the PI3K/AKT/mTOR pathway in the 786-O and ACHN cells of renal cell carcinoma. When the cells were cotreated with 3-MA and sorafenib (or knockdown of the ATG5 gene), the toxic effect of sorafenib was reversed and cell viability was higher when compared to treatment with sorafenib alone [61]. This observation implies that autophagy induced by sorafenib can facilitate cell death in renal cell carcinoma. Interestingly, in our research, flow cytometry was performed for detecting the proportion of apoptotic cells, and the results indicated that the proportion of apoptotic cells in the GW6471 with hydroxychloroquine group was higher than that in the GW6471, hydroxychloroquine alone, and WY14643 with chloroquine groups. With the use of western blotting, we showed that when the 786-O cells were cotreated with GW6471 and hydroxychloroquine, the expressions of cleaved PARP and cleaved caspase-3 were higher than those in the cells treated with GW6471 or hydroxychloroquine alone. Thus, the combined use of GW6471 and hydroxychloroquine decreased the viability of the 786-O cells and promoted apoptosis. This result suggests that inhibiting autophagy can potentiate apoptosis in the 786-O cells treated with GW6471. These results discussed in this paragraph were summarized as a schematic diagram in Figure 7.

Though the role of autophagy in cell death is still unclear, some researchers have explained the relationship between the two with regard to the degradation of the organelles by the autophagosome and the generation of energy by autophagy. Some organelles such as mitochondria and endoplasmic reticulum and lipid droplets can be degraded by autophagosomes [62–64]. Wang et al. reported that gambogic acid can induce apoptosis and autophagy in pancreatic cancer. The level of reactive oxygen species (ROS) was found to increase due to the promotion of damaged mitochondria. It was observed that cotreatment of the pancreatic cells with gambogic acid and chloroquine lowered the cell viability to a greater extent and that the level of ROS was accentuated since the scavenging of the damaged mitochondria was inhibited [65]. In lung cells, perfluoroalkyl acid can decrease cell viability and induce autophagy by engulfing the damaged endoplasmic reticulum and avoiding excessive endoplasmic reticulum stress [66]. On the other hand, energy can be generated via autophagy in the cells under stress. Prostatic cancer is a type of cancer which utilizes lipids for generating energy [67]. Androgen ablation can induce autophagy in androgen-sensitive prostate cancer, and lipid droplets can be degraded via the autophagic pathway for releasing free glycerinum and generating energy for the cells [68]. When autophagy was blocked by knocking down the ATG5 gene, the decrease in the viability of the prostatic cancer cells was higher than that in the androgen ablation group [69]. The ccRCC cells chiefly utilize glucose via glycolysis for generating energy. According to the findings of Aboud et al., GW6471 can inhibit glycolysis via the downregulation of c-Myc in the 786-O cells [39]. Our results showed that GW6471 can enhance autophagy and that cotreatment with GW6471 and hydroxychloroquine can decrease cell viability to a greater extent than treatment with GW6471 alone. Hence, we speculated that hydroxychloroquine inhibited the autophagic flux promoted by GW6471 in the 786-O cells and that the recycling of glucose by autophagy might have been arrested. The glucose content in the 786-O cells cotreated with GW6471 and hydroxychloroquine was insufficient for survival, and the ATP level might have decreased further when compared with the GW6471 alone group, resulting in the aggravation of apoptosis [70].

Hydroxychloroquine has been applied for treating malaria and systemic lupus erythematosus for a long time [71, 72]. In recent years, autophagy has been researched in the field of tumor treatment, and hydroxychloroquine has been applied for treating tumors, including ccRCC, in clinical settings [73]. According to the results of our research, cotreatment with GW6471 and hydroxychloroquine was effective in killing the 786-O renal cell carcinoma cells but had little effect on the HK-2 cells. Along with the results of IHC in ccRCC tissues and the expression of PPAR*α* in the 786-O cell line, our findings imply that the combination of hydroxychloroquine and GW6471 may be beneficial in treating highly differentiated ccRCC, with minimal side effects on normal renal tissues.

We discovered that cotreatment with hydroxychloroquine and GW6471 may be a potentially effective strategy for managing ccRCC. However, we observed that PPAR*α* was poorly expressed in highly differentiated ccRCC tissues and 786-O cell line, which was applied as a cellular model in this research. Hence, our findings may be valid for treating highly differentiated ccRCC only. However, using only one ccRCC cell line for analyzing is the limitation in our research, so we intend to apply more renal cell carcinoma cell lines such as Caki-2 or UM-RC-2 for validating the roles of PPAR*α* in different pathological patterns of RCC.

## 5. Conclusion

Our research has shown that PPAR*α* is poorly expressed in highly differentiated ccRCC tissues and 786-O cells but highly expressed in poorly differentiated ccRCC tissues. Autophagy can be induced by GW6471 in the 786-O cells and blocked by hydroxychloroquine. The combined use of hydroxychloroquine and GW6471 was found to be more effective in killing the 786-O cells than the use of GW6471 or hydroxychloroquine alone, which could be attributed to the promotion of apoptosis. Furthermore, this approach had little effect on the viability of the HK-2 cells, thereby asserting its safety on normal renal tissues. According to the results of this study, the combined use of GW6471 and hydroxychloroquine may serve as a novel and potentially beneficial clinical therapy for highly differentiated ccRCC. However, further *in vivo* studies and clinical trials are required. Moreover, the reason for PPAR*α* agonists not having a significant effect on the proliferation of the 786-O cells and the signaling pathway of autophagy induced by GW6471 in the 786-O cells needs to be explored in the future.

## Figures and Tables

**Table 1:** Characteristics of patients.

Pt. no.	Sex	Age
1	Male	72
2	Female	66
3	Male	55
4	Female	41
5	Male	38
6	Male	46
7	Female	46
8	Male	49
9	Male	52
10	Male	40
11	Male	38
12	Female	57
13	Female	69
14	Male	69
15	Female	53
16	Female	66
17	Female	66
18	Male	59
19	Female	63
20	Male	61
21	Female	60
22	Male	56
23	Female	60
24	Female	37
25	Male	46
26	Male	45
27	Male	60
28	Male	39
29	Female	42
30	Male	44
31	Female	51
32	Female	53
33	Male	61
34	Male	65
35	Female	59
36	Male	57
37	Male	68
38	Female	58
39	Female	64
40	Female	57

**Table 2:** Sequence of primers of GAPDH and PPAR*α*.

Gene	Sequence of upstream primer	Sequence of downstream primer
GAPDH	5′-GCACCGTCAAGGCTGAGAAC-3′	5′-TGGTGAAGACGCCAGTGGA-3′
PPAR*α*	5′-CCATCGGCGAGGATAGTTCTG-3′	5′-CTACATTCGATGTTCAATGCTCCAC-3′

**Table 3:** The procedure of qRT-PCR.

Procedure	Temperature	Time	Cycle
1	94°C	3 min	1
2	94°C	5-10 s	40
	60°C	25 s	

**Table 4:** The relationship between PPAR*α* expression and some clinical factors of ccRCC patients (*n* = 40, chi-square test).

Clinical factors	PPAR*α*		*P* value
	Poorly expression	Highly expression	
	(*n* = 29)	(*n* = 11)	
Sex			0.7271
Male	16	5	
Female	13	6	
Age			0.7235
≤55 years	14	4	
>55 years	15	7	
Differentiation			<0.05
High	27	4	
Poorly	2	7	

## Data Availability

The data used to support the findings of this study are available from the corresponding author upon request.
